# On the correction of spiral trajectories on a preclinical MRI scanner with a high-performance gradient insert

**DOI:** 10.1002/nbm.5249

**Published:** 2024-09-12

**Authors:** Hannah Scholten, Tobias Wech, Sascha Köhler, Sean S. Smart, Jordan H. Boyle, Irvin Teh, Herbert Köstler, Jürgen E. Schneider

**Affiliations:** 1 Department of Diagnostic and Interventional Radiology, University Hospital Würzburg, Würzburg, Germany; 2 Comprehensive Heart Failure Center, University Hospital Würzburg, Würzburg, Germany; 3 Bruker BioSpin GmbH & Co. KG, Ettlingen, Germany; 4 Nuffield Department of Clinical Neuroscience, University of Oxford, Oxford, UK; 5 Faculty of Industrial Design Engineering, Delft University of Technology, Delft, The Netherlands; 6 Leeds Institute of Cardiovascular and Metabolic Medicine, University of Leeds, Leeds, UK

**Keywords:** gradient impulse response, high-performance gradient, preclinical imaging, spiral imaging, trajectory correction

## Abstract

This study aimed to examine different trajectory correction methods for spiral imaging on a preclinical scanner with high-performance gradients with respect to image quality in a phantom and in vivo. The gold standard method of measuring the trajectories in a separate experiment is compared to an isotropic delay-correction, a correction using the gradient system transfer function (GSTF), and a combination of the two. Three different spiral trajectories, with 96, 16, and three interleaves, are considered. The best image quality is consistently achieved when determining the trajectory in a separate phantom measurement. However, especially for the spiral with 96 interleaves, the other correction methods lead to almost comparable results. Remaining imperfections in the corrected gradient waveforms and trajectories are attributed to asymmetrically occurring undulations in the actual, generated gradients, suggesting that the underlying assumption of linearity is violated. In conclusion, images of sufficient quality can be acquired on preclinical small-animal scanners using spiral k-space trajectories without the need to carry out separate trajectory measurements each time. Depending on the trajectory, a simple isotropic delay-correction or a GSTF-based correction can provide images of similar quality.

## 1 Introduction

Magnetic resonance imaging (MRI) has become an indispensable tool in preclinical research, providing non-invasive, multi-parametric and high-resolution images for the study of anatomical, structural, or functional changes in small animal models of human disease. In recent years, there has been a growing interest in refining imaging techniques to enhance both spatial and temporal resolution, crucial, for example, to study cardiovascular disease models.^1^ Among these techniques, spiral k-space trajectories have emerged as a promising method, offering advantages in acquisition speed, signal-to-noise ratio (SNR), and reduced motion and flow artifacts^2–4^ compared to Cartesian image acquisitions. Especially in small animal cardiac MRI, where the heartbeat is very fast and breathing motion cannot be ameliorated by breath hold commands, the acquisition pattern and sampling efficiency of spiral trajectories are favorable. However, there are trade-offs associated with those benefits: Spiral image acquisitions are prone to blurring due to off-resonance effects and to image artifacts caused by trajectory errors.^3,5–9^ The latter arise from spatio-temporal gradient infidelities rooted in partially unavoidable hardware imperfections. In preclinical research settings, where the field strengths are typically higher and the gradient systems are stronger and faster switching than in clinical settings, these limitations become more significant. Spiral k-space trajectories have thus been only sparsely reported in preclinical MRI studies.^3,4,10–17^

Temporal gradient errors arise mainly from uncompensated eddy currents and delays.^18^ Other causes are bandwidth limitations or nonlinear effects of the gradient power amplifiers,^19–21^ and mechanical vibrations of the gradient coils.^22–25^ The most commonly used strategy to mitigate such errors in preclinical imaging is to measure the actual k-space trajectory for each image orientation and set of imaging parameters in a separate experiment.^3,4,15,26,27^ While giving accurate results, this approach can be very time-consuming. Importantly, there are also applications, in which an in situ trajectory measurement is not easily feasible, for example, when employing very high gradient amplitudes, or when the sample is very small or has a very short T2*. In clinical applications, an alternative method based on modelling the gradient system as linear and time-invariant (LTI) has also been established.^5,28–31^ The transmission behavior of the gradient system is characterized in the time domain by the gradient impulse response function (GIRF) or in the frequency domain by the gradient system transfer function (GSTF), either of which can be used to predict the actual trajectories.^5,6,29^ To the best of our knowledge, this approach has so far only been utilized, on preclinical scanners, in hyperpolarized ^13^C imaging,^10^ but not yet in ^1^H imaging. Since the latter comprises a much broader range of applications that could potentially benefit from applying an LTI-model to correct spiral trajectories, we sought to systematically compare trajectory correction methods in preclinical spiral ^1^H MRI.

In this study, we evaluated four correction methods for spiral trajectories on a preclinical small animal scanner with high-performance gradients. The first method was based on measuring the actual trajectories,^26,32^ the second was an isotropic delay correction, the third a gradient correction based on the GSTF, and the fourth a combination of the delay- and GSTF-based corrections. In phantom experiments, we assessed three spiral trajectories with different properties to test the generalizability of the applied correction methods. In in vivo experiments, we targeted cardiac imaging as an application for one of the three spiral trajectories.

## 2 Methods

### 2.1 Hardware

All experiments were performed on a preclinical 7 T small animal scanner (Bruker BioSpec 70/20, Bruker BioSpin GmbH & Co. KG, Ettlingen, Germany) equipped with a high-performance gradient insert (Resonance Research Inc., Billerica MA, USA). The insert has an inner diameter (id) of 60 mm and features a maximum gradient strength of 1.52 T/m and a maximum slew rate of 13,830 T/m/s according to the manufacturer’s specifications. A quadrature-driven birdcage coil (id: 33 mm, Rapid Biomedical GmbH, Rimpar, Germany) was used in transmit-receive mode for all experiments.

### 2.2 Phantoms

GSTF measurements were performed on a plastic sphere (outer diameter [od]: 29 mm) filled with distilled water. Phantom images were acquired on a 50-mL Falcon tube (od: 29 mm, length: 116 mm) with plastic building bricks inside, which were embedded in agarose gel (1%), doped with 0.1% CuSO4.

### 2.3 Spiral imaging experiments

**Figure 1:**
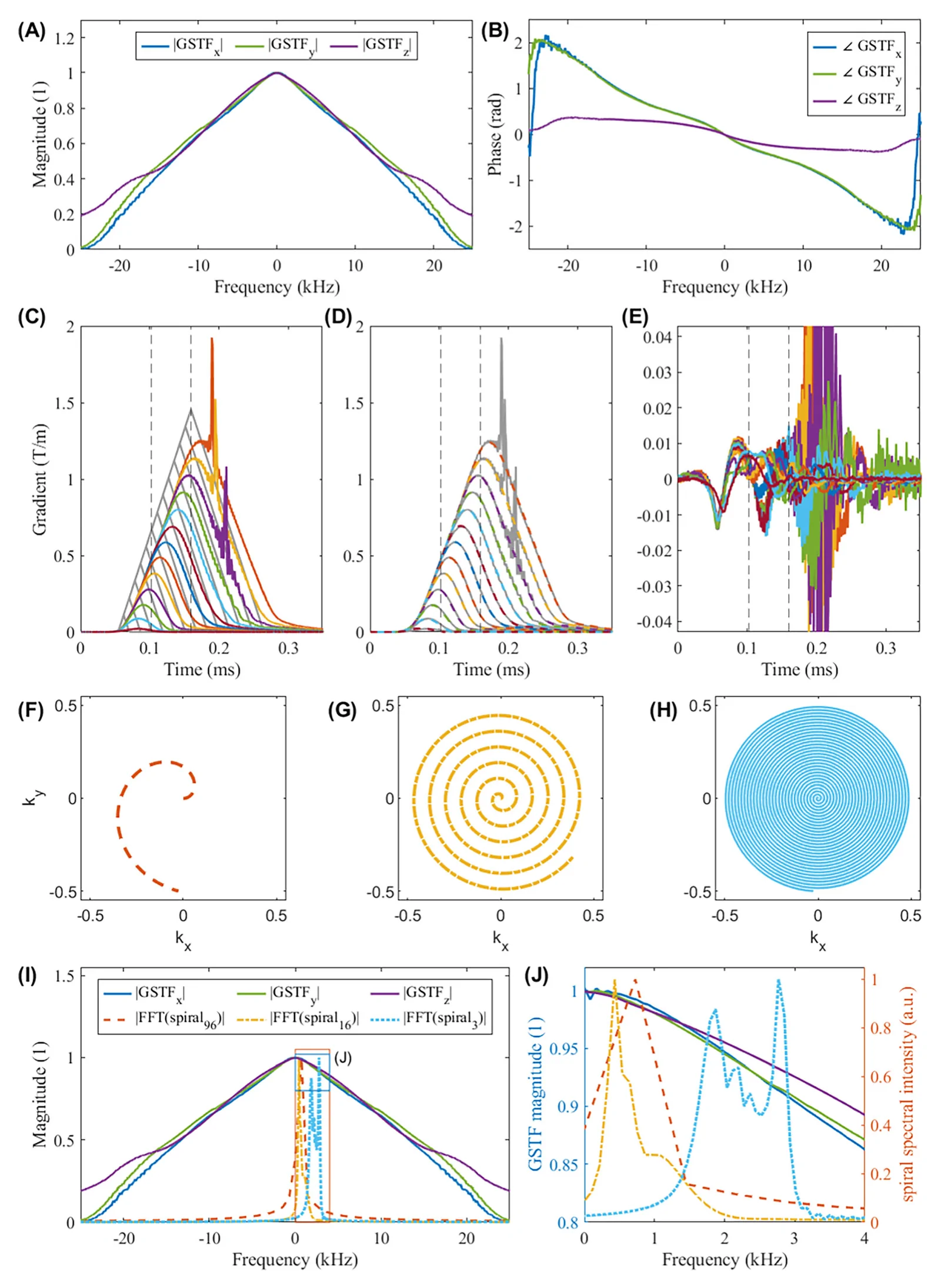
(A) Magnitude and (B) phase of the gradient system transfer functions of the *x*-, *y*-, and *z*-axis. (C) Nominal (gray) and measured (colored) waveforms of the triangles used for the GSTF calculation on the *x*-axis. The artifacts in the three largest measured triangles are caused by inaccurate estimates of the signal phase due to too much dephasing. The dashed vertical lines mark the peaks of the 6th and 13th triangles in the nominal waveforms. (D) Measured (gray, solid) and calculated (colored, dashed) waveforms of the triangles. (E) Differences between the measured and calculated waveforms. (F) Spiral trajectory with 96 interleaves. (G) Spiral trajectory with 16 interleaves. (H) Spiral trajectory with three interleaves. (I, J) magnitudes of the GSTFs and of the spectra of the three spiral waveforms used in the phantom experiments. GSTF, gradient system transfer function.

We acquired phantom images with three different Archimedean spiral trajectories, using the vendor-provided spiral imaging sequence. Trajectory 1 consisted of 96 interleaves, trajectory 2 consisted of 16 interleaves, and trajectory 3 of three interleaves. They are depicted in Figure 1. The spiral gradients are designed under constraint of a maximum slew rate and an amplitude limit, and optionally an additional frequency limit. From the trajectories resulting with and without the frequency limit, the sequence algorithm automatically chooses the shorter one. We chose trajectories with different readout lengths and bandwidths in order to investigate how these parameters affect the quality of the applied correction methods. The imaged slice was positioned at the isocenter of the magnet in a double-oblique orientation.

In vivo proof of concept experiments were performed using trajectory 1 in ~22-week-old C57Bl6/J mice (male, 31.13 ± 0.76 g, *n* = 3). Mice were anesthetised with 4% isoflurane in 100% medical oxygen and maintained on a nose cone at 1.5–2% throughout. Mice were placed prone on a temperature-controlled cradle.^33^ After scouting for short- and long-axis views, double-gated (i.e., using electrocardiogram [ECG]-triggering and respiratory gating) cine experiments with steady-state maintenance were performed as previously described,^34^ but using a flexible, programmable gating controller.^35^ Cartesian and spiral images were acquired in an interleaved fashion covering the heart from base to apex. All animal experiments were approved under project license PP5900882 by the Committee for Animal Care and Ethical Review at the University of Leeds and comply with the UK Animals (Scientific Procedures) Act 1986, as amended 2012.

All imaging parameters are summarized in Table 1. The Cartesian in vivo images were acquired with fewer averages than the spirals in order to keep the number of incorporated cardiac cycles the same. Images with 16 or three spiral interleaves were not acquired in vivo because we wanted to match the temporal resolution of the Cartesian cine acquisition of 4.8 ms.^34^ This was not achievable with 16 or three interleaves, even with higher bandwidth.

### 2.4 Trajectory measurements

The trajectories were measured with the thin-slice method^26,32^ immediately before the respective image acquisition. In short, the k-space position along a physical gradient axis is deduced from the phases of multiple free induction decay (FID) signals. These signals are measured for every gradient axis of interest in two thin, coplanar off-center slices (slice thickness: 0.16 mm, slice positions: ±4.5 mm from isocenter, slice orientation: perpendicular to the gradient axis of interest) while the projection of the imaging gradient to the respective axis of interest is active. Reference measurements without the gradient of interest are recorded to remove effects of the slice selection gradient from the signals. Considering a double-oblique slice orientation, where the spiral gradients have components on all three gradient axes, three thin slice measurements need to be conducted. In total, two FID measurements (one with the respective spiral gradient component active and one reference without it) in six slices each (two perpendicular to the *x*-, *y*-, and *z*-axis, respectively) are needed to determine the actual trajectory for each spiral interleaf. The trajectory measurements applied the same bandwidth, number of readout points, and repetition time as the image acquisitions. Thus, 12 times the time of a single spiral image acquisition was added to each double-oblique imaging experiment.

For the in vivo measurements, we performed two separate trajectory measurements. One in the mouse, but without any gating, and one in the spherical phantom filled with distilled water. In the gated acquisition, the trajectory measurement was not possible with the sequence we used, seemingly due to the software version on the scanner.

### 2.5 GSTF measurements and calculation

The GSTFs of the three physical gradient axes were determined from 13 triangular test gradient pulses. These were also measured with the thin-slice method^26,32^ for each axis (slice thickness: 0.2 mm, slice positions: ±5.1 mm, receiver bandwidth: 1000 kHz, readout length: 50 ms, TR: 10 s). One reference FID signal without any active gradients was acquired per slice to compensate effects from the slice selection gradient. For the GSTF measurements, we also used the spherical water phantom. Each GSTF was measured with 32 averages. The 13 triangles featured ramp times between 8.1 and 105.3 μs, with an increment of 8.1 μs, which equals the gradient raster time of the scanner. We chose a slew rate of 13,827.2 T/m/s, which is close to the maximum possible slew rate. The triangular waveforms thus reached maximum amplitudes between 0.112 and 1.456 T/m. The three test gradients with longest duration had to be excluded from the calculation of the GSTF because the induced dephasing did not allow to determine the phase of the signal accurately on the falling edge of the triangles.

We calculated the GIRF, i.e., the Fourier-transform of the GSTF, in the time domain through a linear system of equations. These were implemented as a matrix equation^36^ and solved using Tikhonov regularization^37,38^:

(1) $$\left[\begin{array}{c} g_{\text{out}\;}\\ 0 \end{array}\right]=\left[\begin{array}{cc} M_{\text{in}\;} & 1\\ \lambda R & 0 \end{array}\right]\left[\begin{array}{l} h\\ \Delta \end{array}\right]\;\text{with}\;R=I$$

The column vector **g**_out_ holds the *N*_out_ measured gradient samples; the column vector h consists of the *N*_h_ discretized values of the GIRF; the *N*_out_ × *N*_h_ matrix M_in_ contains shifted instances of the input waveform, implementing the discretized convolution of the input waveform with the GIRF by the product of M_in_ and h; 1 represents an *N*_out_ × 1 vector of ones; the scalar Δ symbolizes a constant gradient field offset; λ = √0:1 is the scaling factor for the *N*_h_ × *N*_h_ regularization matrix R, which we chose to be equal to the identity matrix I. This calculation approach was chosen because it results in less noisy GSTFs than the direct calculation in the frequency domain,^38^ and it is less prone to artifacts in the resulting GSTFs arising from inaccuracies in the measured gradient waveforms **g**_out_.^36^

In order to reduce the computational demand and further reduce noise in the GSTF, we only took into account the first 12 ms of each readout, resulting in a frequency resolution of 83.3 Hz. In a prior calculation with a frequency resolution of 20 Hz, we verified that no mechanical resonances or other long-living gradient distortions are present on our system.

### 2.6 Image reconstruction

For non-Cartesian image reconstruction, we used the non-uniform FFT (NUFFT) toolbox^39^ included in the Michigan Image Reconstruction Tool-box (MIRT).^40^ Each spiral image was reconstructed five times, each with a different k-space trajectory. The first reconstruction used the prescribed trajectory. The second reconstruction used an isotropically delay-corrected trajectory: the prescribed gradient waveforms on all three axes were delayed by the same amount of time, and then integrated. The third reconstruction used the trajectory as predicted by the GSTF, which was calculated by multiplying the Fourier-transformed gradient progression of each spiral interleaf on each axis with the respective self-term of the GSTF.^30^ Since the GSTFs did not reveal any long-living gradient distortions, it was justified to treat every repetition time (TR) interval independently from the previous TRs for this calculation. For the fourth reconstruction, a small, additional isotropic delay was applied to the GSTF-corrected gradient waveforms (“GSTF + delay” correction). This was done to reduce residual artifacts in the GSTF-corrected images and will be further explained in the discussion of this article. Finally, for the fifth reconstruction, the trajectory was measured with the thin-slice method, as described above.

The optimum delays for the delay- and the GSTF + delay correction were determined by comparing the images to the ones reconstructed with the measured trajectory, which we considered as ground truth images. Images were reconstructed with different delays, and a normalized root-mean-squared error (nRMSE) was calculated for each delay *τ*, according to

(2) $$\mathrm{nRMSE}(\tau )=\frac{\sqrt{\sum_{i=1}^{N^{2}}{\left|S_{i}^{\text{meas}\;}-a\cdot S_{i}^{\text{delay}\;}(\tau )\right|}^{2}}}{N}$$

(3) $$\;\text{with}\;a=\frac{\sum_{i=1}^{N^{2}}\left|S_{i}^{\text{meas}\;}\right|\cdot \left|S_{i}^{\text{delay}\;}(\tau )\right|}{\sum_{i=1}^{N^{2}}{\left|S_{i}^{\text{delay}\;}(\tau )\right|}^{2}}\text{.}\;$$

$S_{i}^{\text{meas}\;}$ and $S_{i}^{\text{delay}\;}$ are the image intensities in the *i*^th^ pixel of the magnitude images reconstructed with the measured and the corrected trajectory, respectively, and *N*^2^ signifies the number of pixels. The normalizing factor *a* as defined in Equation (3) accounts for intensity differences in the two images. We then fitted a polynomial of fourth degree to the outcome and determined the minimum by finding the roots of the first derivative.

### 2.7 Simulations in a numerical phantom

To better understand how trajectory errors and the various corrections manifest in the resulting images, we also performed simulations in a numerical phantom. We produced synthetic k-space data by applying the forward NUFFT operator of each measured spiral trajectory to a modified Shepp–Logan phantom. Subsequently, we reconstructed the image with each of the (un-)corrected trajectories that we also used for the reconstruction of our measured phantom images. Changes or artifacts in the resulting images would then only be caused by the trajectory errors, allowing us to assess whether those were the dominating effect in our experiments. Otherwise, other confounding factors, such as static *B*_0_ inho-mogeneity, could have potentially played a role.

## 3 Results

### 3.1 GSTFs of the physical gradient axes

Figure 1 displays the GSTFs of the *x*-, *y*-, and *z*-axis of the high-performance gradient system in the top row. The magnitude (Figure 1(A)) exhibits a low-pass characteristic that looks similar on all three gradient axes in the range up to 15 kHz. Outside the range (−15 kHz, 15 kHz), GSTF_*z*_ is wider than the other two. The phase (Figure 1(B)) of GSTF_*x*_ and GSTF_*y*_ exhibits multiple bends and a negative slope, and is almost identical for the *x*- and *y*-axis, while the slope of the phase of GSTF_*z*_ is much flatter.

The second row shows the triangles that were used to calculate the GSTF on the *x*-axis. Figure 1(C) shows the nominal (gray lines) and measured waveforms (colored lines). The measured triangles with the three largest areas are confounded by artifacts on the falling edge, caused by too much dephasing of the signal. When the amplitude of the FID signal is zero, the phase can no longer be determined accurately, in this case leading to erroneous gradient estimates. These three triangles were therefore excluded from the GSTF calculation. The dashed vertical lines mark the peaks of the 6th and 13th nominal triangle waveforms as reference points in Figure 1(C, D, and E). Figure 1(D) displays the measured wave-forms (gray solid lines) again, as well as the ones calculated by GSTF_*x*_ (colored dashed lines). Except for the confounded parts of the three largest triangles, the measured and calculated curves are indistinguishable by eye. The differences are plotted in Figure 1(E). From the differences, it can be seen that the measured gradient is always smaller than the prediction immediately after switching on the gradient. It then rises above the predicted values, before falling onto or below the prediction again. The undulation occurring before the first dashed reference line is very similar for all triangles except the first four, which pass their respective peak values before this point. For all the remaining triangles, this undulation presents on the rising edge, but is not mirrored symmetrically on the falling edge. Instead, the measured waveforms show slight oscillations around the predicted ones on the falling edges of the triangles. The large deviations around 0.2 ms in the green (10th triangle) and light blue (9th triangle) graphs in Figure 1(E) are caused by measurement noise.

The third row presents one interleaf each of the three spiral trajectories that were used in the phantom experiments, namely with 96 (Figure 1 (F)), 16 (Figure 1(G)), and three interleaves (Figure 1(H)).

In the bottom row of Figure 1, the magnitudes of the GSTFs are overlaid by the spectra of the gradient waveforms corresponding to the three spiral trajectories used in the phantom experiments. The spiral waveform with 16 interleaves has the lowest main frequency range, with the highest peak of the spectrum at 0.43 kHz. The spiral with 96 interleaves exhibits a wider main peak with the maximum at 0.73 kHz. The spectrum of the spiral with three interleaves contains an even wider range of frequencies in the main lobe, and shows two prominent peaks at 1.87 and 2.77 kHz. The zoomed view in Figure 1(G) reveals that there are no resonances present in any of the GSTFs in the relevant frequency range.

### 3.2 Delay optimization

**Figure 2:**
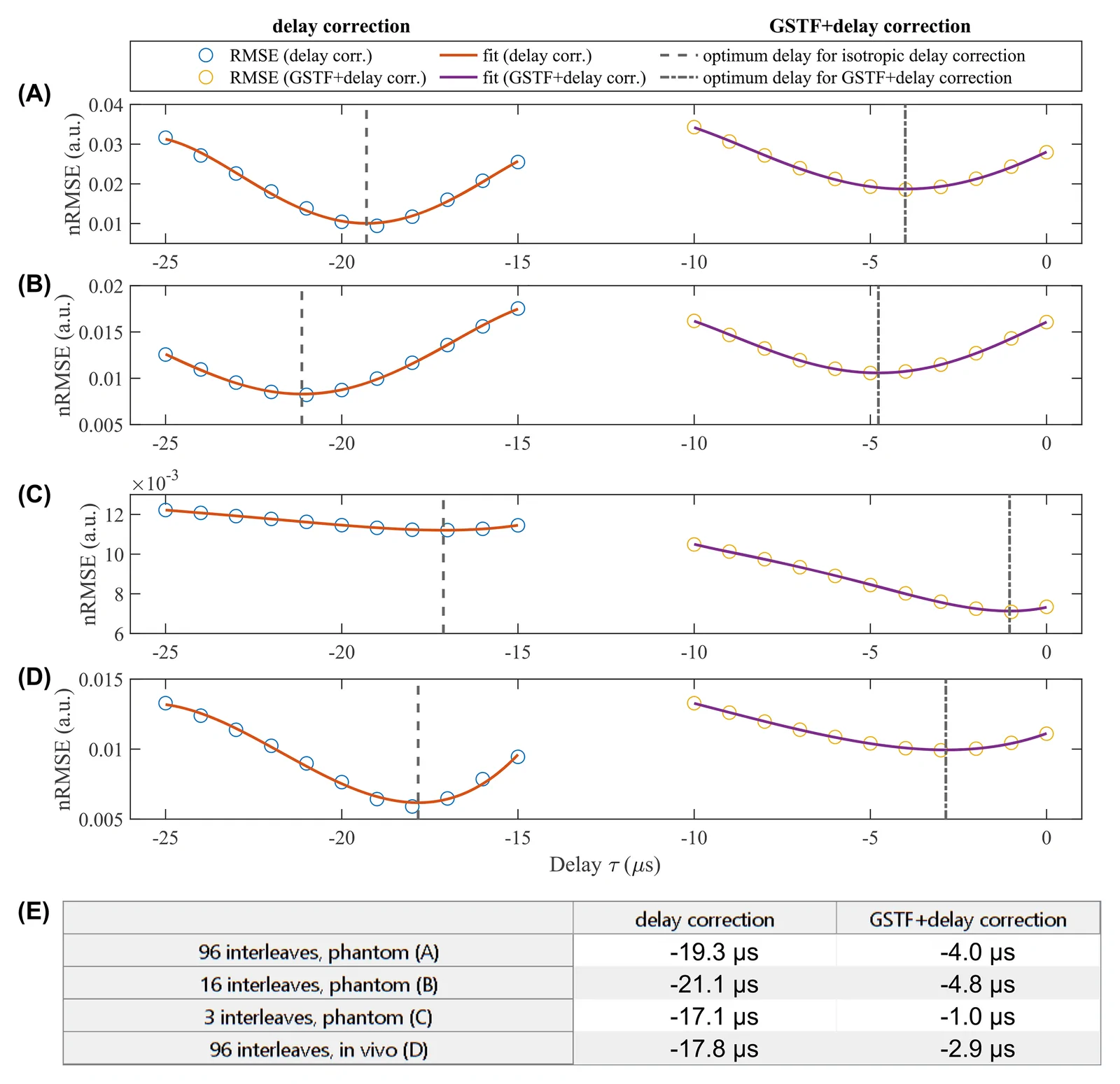
Delay optimization for the isotropic delay correction (left) and the GSTF + delay correction (right). (A) Phantom image with 96 spiral interleaves. (B) Phantom image with 16 spiral interleaves. (C) Phantom image with three spiral interleaves. (D) Mouse image with 96 spiral interleaves. (E) Optimum delays as marked by the vertical lines in (A–D). GSTF, gradient system transfer function.

Figure 2 shows the nRMSEs (according to Equation (2)) of the different spiral reconstructions for different values of the delay τ in both the trajectory correction with the isotropic delay and in the GSTF + delay trajectory correction, respectively. In all cases, the minimum for the isotropic delay correction was found to range between −17 and −22 μs, while the optimal delay for the GSTF + delay correction was between 0 and −5 μs. The exact values are summarized in Figure 2(E). Notably, the delays of acquisitions with different imaging parameters are different, but also the delays of both acquisitions with 96 spiral interleaves, i.e., in the phantom and in vivo, differ, which will be addressed in the discussion.

### 3.3 Phantom images

**Figure 3:**
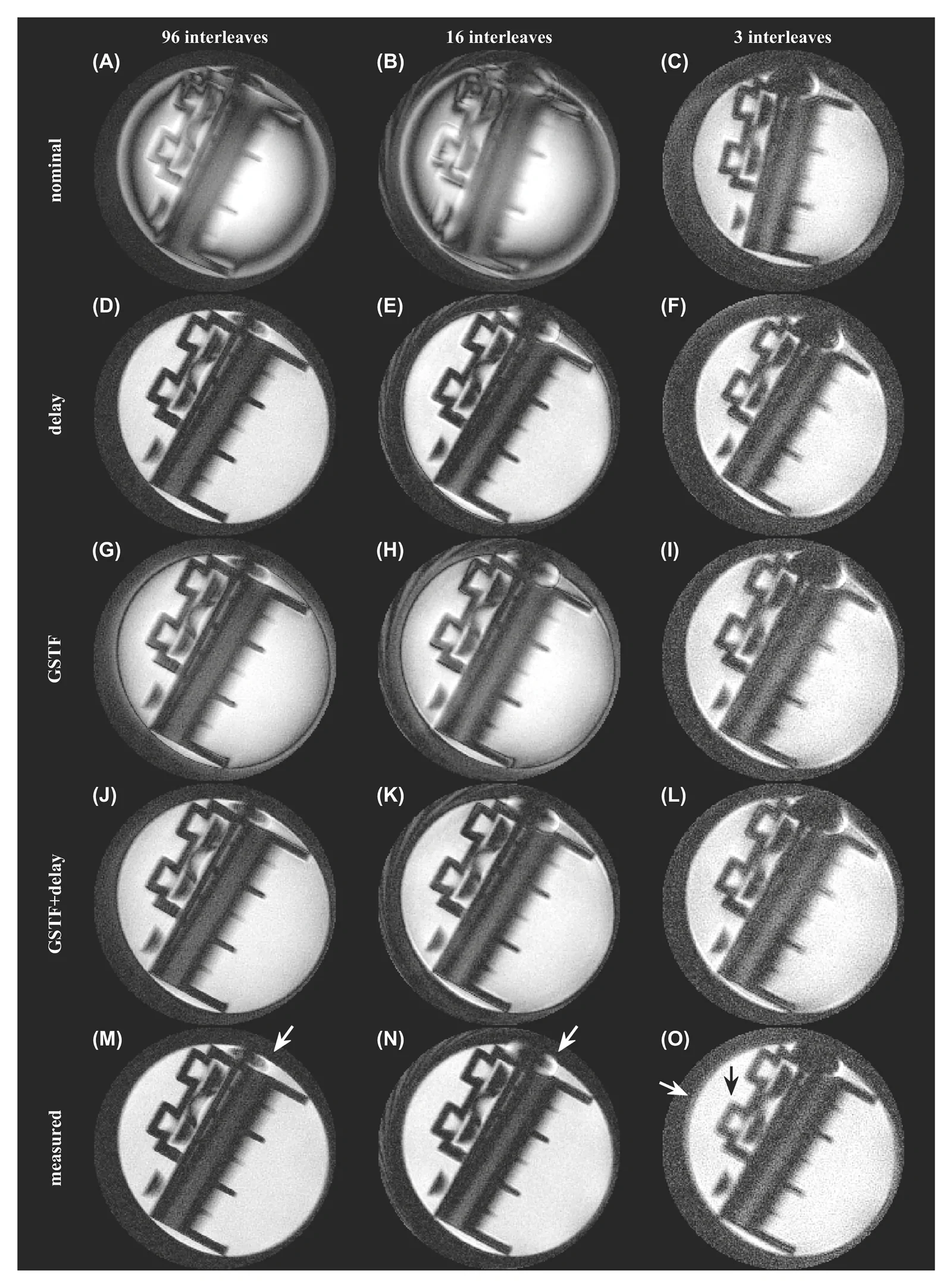
Phantom images acquired with three different spiral trajectories. Column 1: spiral with 96 interleaves. Column 2: spiral with 16 interleaves. Column 3: spiral with 3 interleaves. Row 1: reconstruction with the nominal trajectory. Row 2: reconstruction with the delay-corrected trajectory. Row 3: reconstruction with the GSTF-corrected trajectory. Row 4: reconstruction with the GSTF + delay-corrected trajectory. Row 5: reconstruction with the measured trajectory. The arrows indicate artifacts caused by an air bubble and blurry parts of the phantom, as mentioned in the text.

Figure 3 shows the phantom images acquired with 96, 16, and three spiral interleaves, each reconstructed with five different trajectories.

For the spirals with 96 and 16 interleaves, the reconstruction with the nominal trajectory yields significant artifacts in the images (Figure 3(A, B)). For the spiral with three interleaves, the rim of the phantom is uneven, and the center appears brighter compared to the outer region (Figure 3(C)). The delay-corrected trajectory works well for the spiral with 96 interleaves (Figure 3(D)), but still suffers from hyperintense artifacts around the edges of the building brick for the spiral with 16 interleaves (Figure 3(E)). For the spiral with three interleaves, the delay-correction seems to just rotate the image and alter the intensity distribution slightly (Figure 3(F)), compared to the nominal reconstruction. Using the GSTF-corrected trajectory, we see hyperintensities around the edges of the building brick in the spiral images with 96 and 16 interleaves (Figure 3(G, H)). Additionally, there is a halo around the rim of the phantom, and the image intensity is highest in the center and decreases towards the edges. With the GSTF + delay correction, these artifacts are reduced significantly (Figure 3(J,K)). Comparing the central low SNR region of the phantom in Figure 3(G, H, J, and K) to Figure 3(M and N), it appears slightly larger in the former. For the spiral with three interleaves, both GSTF-corrected images (Figure 3(I,L)) look similar to the delay-corrected image, except the whole phantom appears larger. The reconstruction with the measured trajectory results in almost artifact-free images for the spirals with 96 and 16 interleaves (Figure 3(M,N)). The only visible artifacts come from an air bubble enclosed in the phantom about the 1 o’clock position (indicated by arrows). The image reconstructed with the measured trajectory of the spiral with three interleaves exhibits only little artifacts from the air bubble, but appears blurry between 9 and 11 o’clock, indicated by the arrows in Figure 3(O).

Among the different reconstructions for each spiral configuration, the one with the measured trajectory always yields the best image quality. For the spiral trajectory with 96 interleaves, the reconstructions with the delay-correction, the GSTF + delay correction, and with the measured trajectory are of similar image quality with little to no visible artifacts. For the spiral with 16 interleaves, the image with the GSTF + delay correction (Figure 3(K)) only shows a slightly hyperintense rim at the external border of the phantom and the signal intensity within the phantom is not as homogeneous as in the image with the measured trajectory. For the spiral with three interleaves, all the images suffer from high noise levels, which also make the inner structure of the phantom look blurry. However, the reconstruction with the measured trajectory looks least distorted around the air bubble and has the most even image intensity distribution in the agarose part of the phantom, despite potentially being a bit noisier.

**Figure 4:**
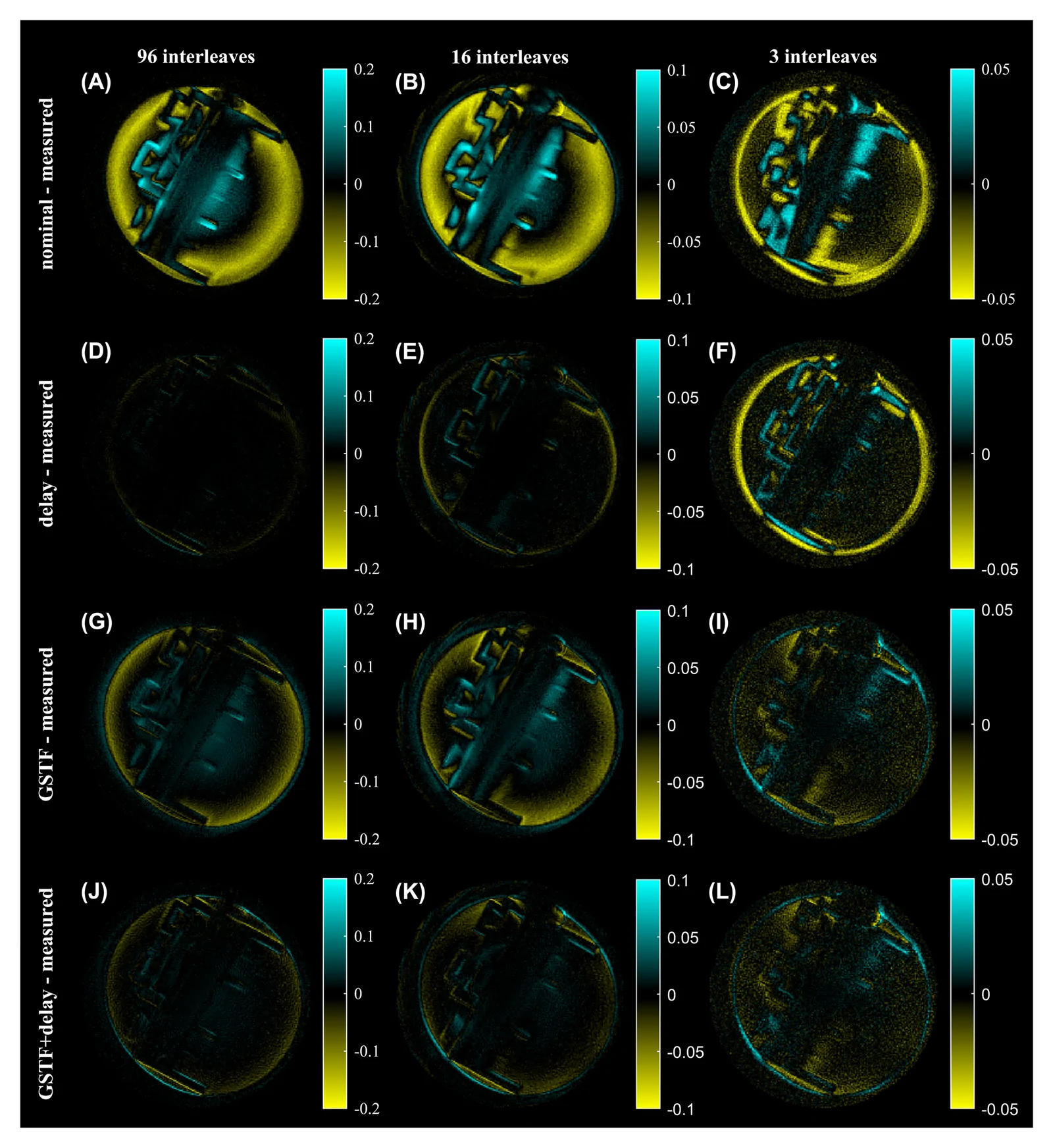
Difference images between the phantom reconstructions with the nominal (row 1), delay-corrected (row 2), GSTF-corrected (row 3), GSTF + delay-corrected (row 4), and with the measured trajectories. Column 1: spiral with 96 interleaves. Column 2: spiral with 16 interleaves. Column 3: spiral with 3 interleaves. GSTF, gradient system transfer function.

Figure 4 depicts difference images between the phantom reconstructions with the nominal, delay-corrected, GSTF-corrected, GSTF + delay-corrected, and with the measured trajectories to examine the geometric congruence of the structure in the phantom more closely. In spiral images, trajectory errors that resemble a delay result in a rotation of the imaged object, so checking the geometry provides another means of evaluating the trajectory corrections we applied. The maxima of the colormaps are the same as in the respective columns in Figure 3. For the trajectory with 96 interleaves (Figure 4, column 1), the delay-corrected reconstruction shows the least deviations from the “measured” one (Figure 4 (D)), closely followed by the GSTF + delay-corrected reconstruction (Figure 4(J)). For the GSTF-corrected reconstruction, the difference image (Figure 4(G)) demonstrates mostly intensity differences compared to the ground truth image, but no significant geometric differences. Specifically, the image intensity is higher in the center and lower towards the edges of the phantom, which might cause or contribute to the impression described previously, that the central part of the phantom appears larger. For the trajectory with 16 interleaves (Figure 4, column 2), the GSTF-corrected reconstruction (Figure 4(H)) shows the same intensity differences, but with an additional slight rotation of the phantom. The delay-corrected and the GSTF + delay-corrected phantom images (Figure 4(E and K)) exhibit minor geometric misalignments, in the latter case accompanied by small intensity differences. For the trajectory with three interleaves, Figure 4(C and F) clearly demonstrate that the nominal trajectory and the isotropic delay correction result in the phantom appearing too small. Figure 4(F) also proves that the delay correction rotates the phantom into the correct orientation. The GSTF correction (Figure 4(I)) yields an image where the phantom’s size is represented correctly, with only minor geometric infidelities remaining. They are slightly ameliorated with the GSTF + delay correction (Figure 4(L)), but the visual impression that the additional delay does not change the image significantly is confirmed.

**Figure 5:**
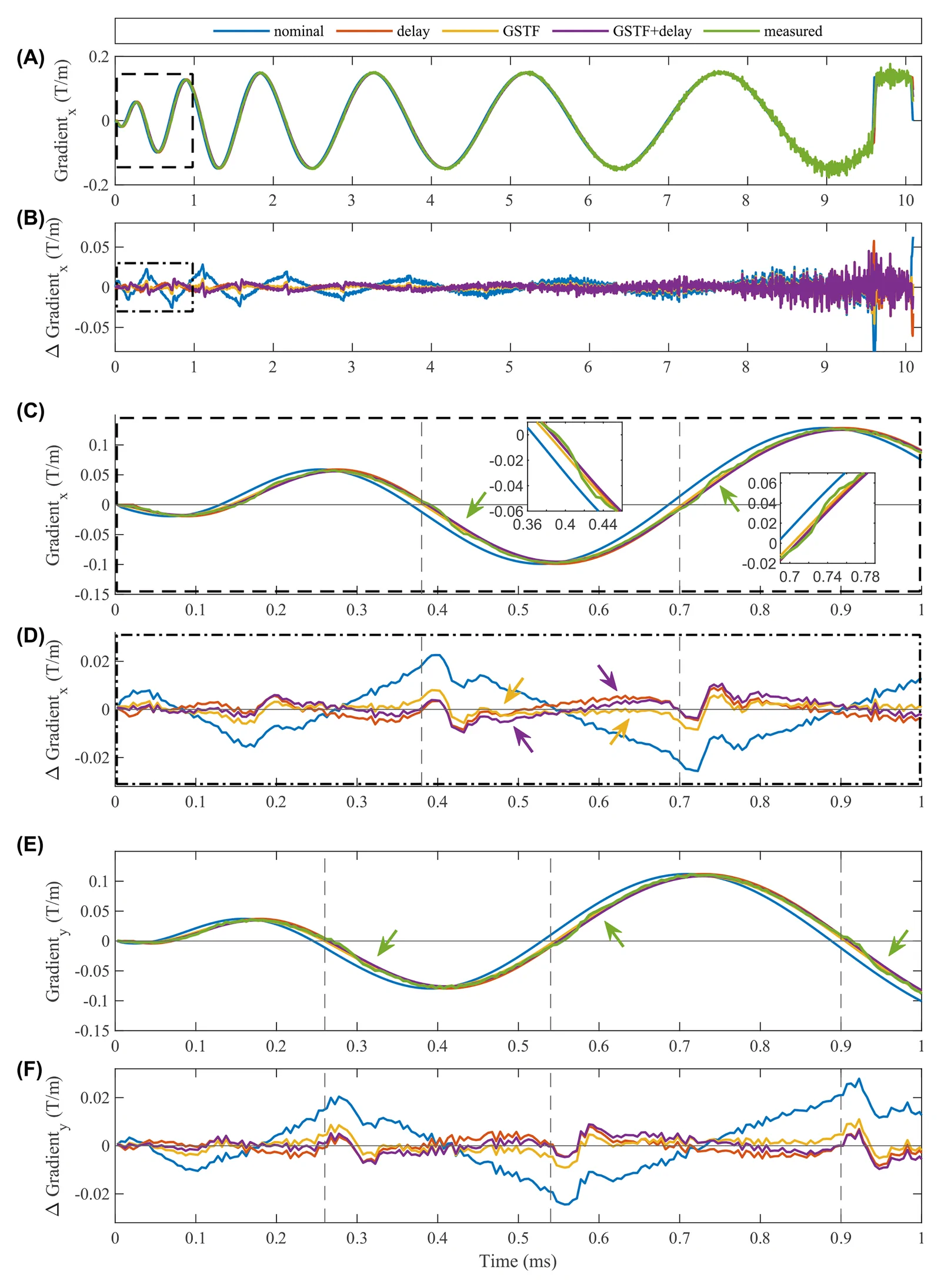
Gradient waveforms of one spiral interleaf of the phantom measurement with the spiral with 16 interleaves. (A) Gradient waveforms without and with the different corrections on the *x*-axis of the gradient system. (B) Differences of the nominal and corrected gradient waveforms to the measured one. (C) Zoom into the first millisecond of the gradient waveforms. (D) Zoom into the first millisecond of the differences. (E) First millisecond of the gradient waveforms on the *y*-axis of the gradient system. (F) First millisecond of the differences of the nominal and corrected waveforms to the measured one on the *y*-axis.

Figure 5 displays gradient waveforms of the phantom measurement with 16 interleaves. Figure 5(A) illustrates the waveform on the gradient *x*-axis of one spiral interleaf, while Figure 5(B) shows the differences of the nominal and corrected waveforms to the measured one. The nominal waveform clearly differs most from the measurement. Figure 5(C,D) zoom into the first millisecond, allowing to examine more subtle differences. The nominal gradient waveform is obviously shifted against the other ones. Differences between the delayed and the two GSTF-corrected wave-forms are not depicted well in the chosen visualization in Figure 5(C), but the measured gradient waveform exhibits small undulations on the left slope of each lobe (marked by the green arrows and zoomed-in insets), which do not appear on the respective right slopes. Notably, these asymmetric undulations are not present in any of the calculated waveforms. The dashed lines mark the start and end of the third sinusoidal lobe of the waveform, facilitating the comparison of the gradient waveforms in Figure 5(C) to the differences to the measured one in Figure 5(D). In the first 0.15 ms in Figure 5(D), the GSTF + delay-corrected gradient waveform exhibits the least deviations from the measured waveform (as the depicted difference is almost zero). From 0.15 ms onwards, the GSTF-corrected waveform without the additional delay seemingly agrees best with the measured one, as the difference only deviates from zero when the undulations in the measured waveform occur. The arrows in Figure 5 (D)exemplarily highlight time points in between the undulations where the GSTF + delay-corrected waveform deviates more from the measured one than the GSTF-corrected waveform without the additional delay. However, over the course of the whole image acquisition, taking into account all spiral interleaves, the RMSE between the measured and GSTF + delay-corrected waveform is lower than the RMSE between the measured and GSTF-corrected waveform (data not shown). This applies to all three gradient axes. Figure 5(E,F) show the first millisecond of the respective gradient waveform on the *y*-axis, where similar observations can be made.

**Figure 6:**
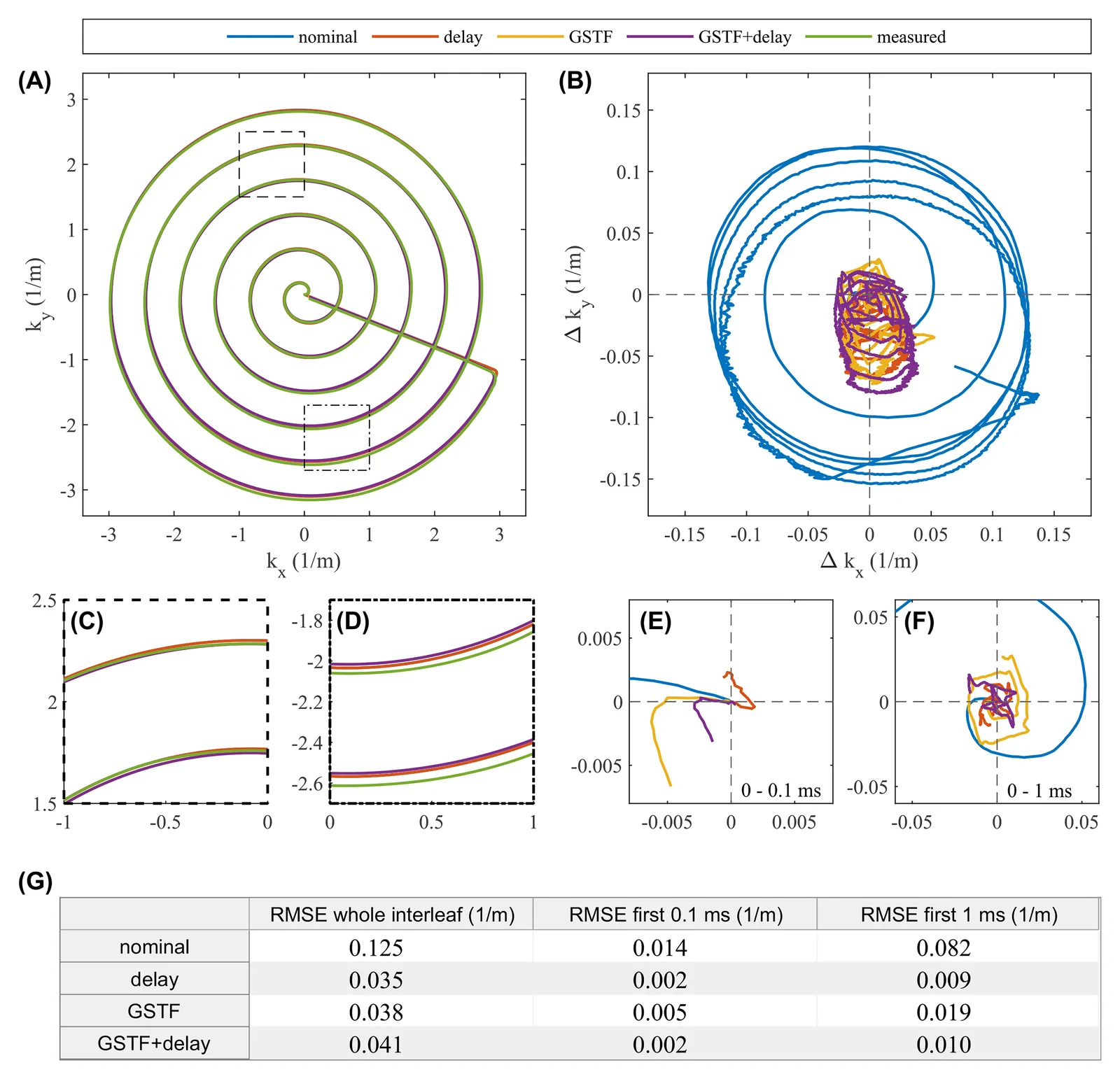
K-space trajectories of one spiral interleaf of the phantom measurement with the spiral with 16 interleaves. (A) Nominal, corrected, and measured k-space values. (B) Differences of the nominal and corrected trajectories to the measured one. (C) Zoom onto the trajectories in the upper half of k-space. (D) Zoom onto the trajectories in the lower half of k-space. (E) Trajectory differences of the first 0.1 ms of the readout. (F) Trajectory differences of the first millisecond of the readout. (G) RMSEs corresponding to the differences in (B) (column 1), (E) (column 2), and (F) (column 3). RMSEs, root-mean-squared errors.

Figure 6 compares one interleaf of the k-space trajectories of the different reconstructions of the phantom measurement with 16 interleaves. In Figure 6(A), it can be seen that all five trajectories lie closely together in the upper half of k-space, while there is a distinct deviation of the measured trajectory from the other four in the lower half of k-space. The zoomed-in sections in Figure 6(C,D) illustrate this more clearly. The differences between the measured and the other four trajectories are depicted in Figure 6(B). The nominal trajectory obviously deviates most from the measured one. Zooming into the k-space center in Figure 6(E,F), differences for the first 0.1 ms and the first millisecond of the readout are evident. The RMSEs corresponding to the differences plotted for the different time windows are summarized in Figure 6(G). Notably, the RMSE of the GSTF-corrected trajectory is more than twice as large as the RMSE of the delay- and GSTF + delay-corrected trajectories in the first 0.1 ms. This matches the visual impression in Figure 3, where the intensity distribution of the GSTF-corrected image differs notably from that in the images with the delay- and GSTF + delay correction. The situation in the time window of the first millisecond is comparable.

### 3.4 Simulations in a numerical phantom

**Figure 7:**
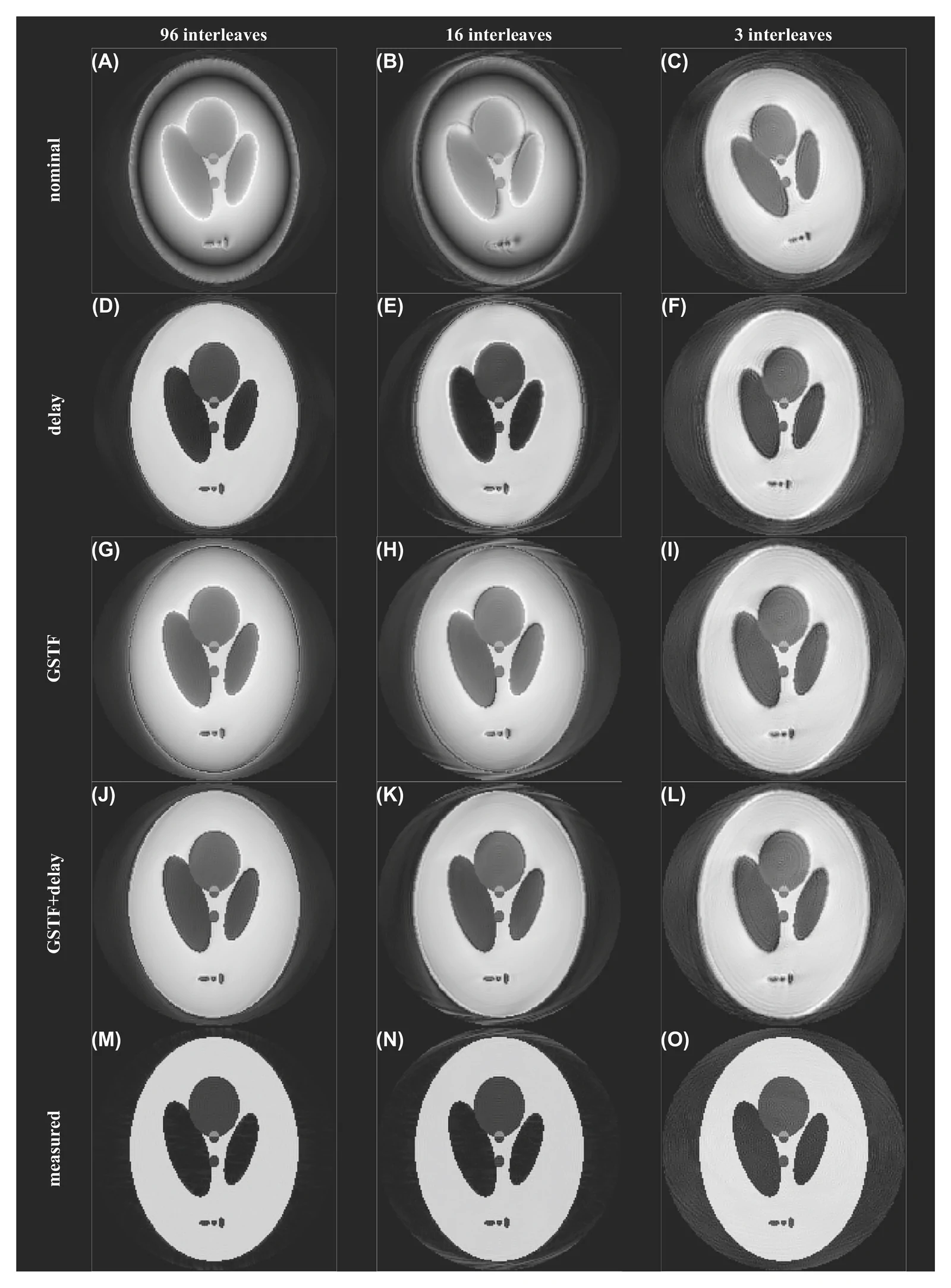
Reconstructions of the synthetically sampled Shepp-Logan phantom. Column 1: spiral with 96 interleaves. Column 2: spiral with 16 interleaves. Column 3: spiral with 3 interleaves. Row 1: reconstruction with the nominal trajectory. Row 2: reconstruction with the delay-corrected trajectory. Row 3: reconstruction with the GSTF-corrected trajectory. Row 4: reconstruction with the GSTF + delay-corrected trajectory. Row 5: reconstruction with the measured trajectory, i.e. the adjoint of the NUFFT operator that was used to create the synthetic k-space data.

The reconstructions of the synthetically sampled Shepp–Logan phantom are shown in Figure 7, in the same order as the phantom images in Figure 3. The artifacts described previously for the actual phantom MR images, i.e., intensity variations, hyperintense rims, halos, rotation, and geometric scaling, are also present in a very similar manner in the numerical simulations. Given the striking resemblance between the two sets of images, we believe a detailed description would be redundant.

### 3.5 In vivo images

**Figure 8:**
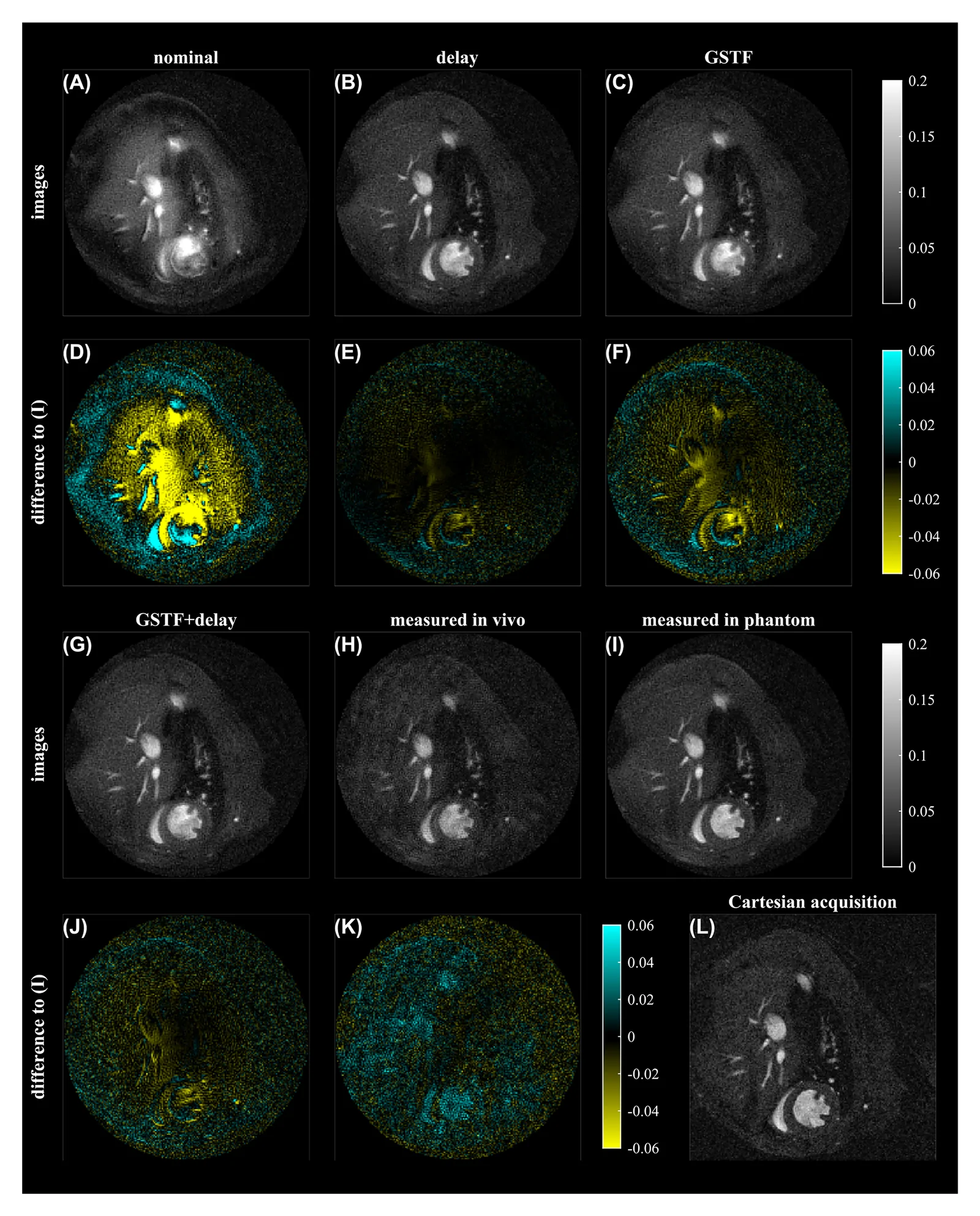
Short axis view of a mouse’s heart. (A, B, C, G, H, I): spiral images reconstructed with different trajectories, as indicated in the figure. (D, E, F, J, K): difference images of the different reconstructions to the ground truth image (I). (L) Cartesian image.

Figure 8 shows the short axis view of a murine heart, reconstructed with different trajectory corrections. In contrast to the phantom measurements, two of the in vivo images were reconstructed with a measured trajectory. The trajectory was measured both in situ (Figure 8(H)), and in a separate phantom measurement (Figure 8(I)). The latter image was considered the ground truth image. Figure 8 also shows the difference between each reconstructed image and the ground truth. A Cartesian image is shown in Figure 8(L) for comparison. The center of the image is hyperintense in the reconstruction with the nominal trajectory (Figure 8(A)), whereas hypointense regions can be seen in the outer parts of the field of view (FOV). The myocardium and the blood pool cannot be distinguished due to blurring and smudging artifacts. As far as the reconstructions with the delay-, GSTF-, and GSTF + delay-corrected trajectories are concerned, it is hard to make out differences to the ground truth image by eye. The difference images (Figure 8(E, F, J)) reveal that the image with the isotropic delay correction (Figure 8(B)) agrees well with the ground truth image. In the two GSTF-corrected images (Figure 8(C, G)), the signal intensity is slightly too high in the center and too low in the periphery of the FOV. This is more pronounced when the GSTF correction is performed without the additional delay. In all three cases, the differences are largest in the heart. In the image where the trajectory was measured in vivo (Figure 8(H)), we see random intensity variations over the entire FOV, which make the image look noisier than the other ones. On the other hand, measuring the trajectory separately in a phantom yields a sharp image without any visible artifacts (Figure 8(I)). The Cartesian acquisition appears to be artifact-free, too, but the gray scale in Figure 8(L) is different from the other images due to the different reconstruction (inverse FFT instead of NUFFT). The upper limit of the color scale was chosen such that the image appears similar in image intensity to the spiral images.

**Figure 9:**
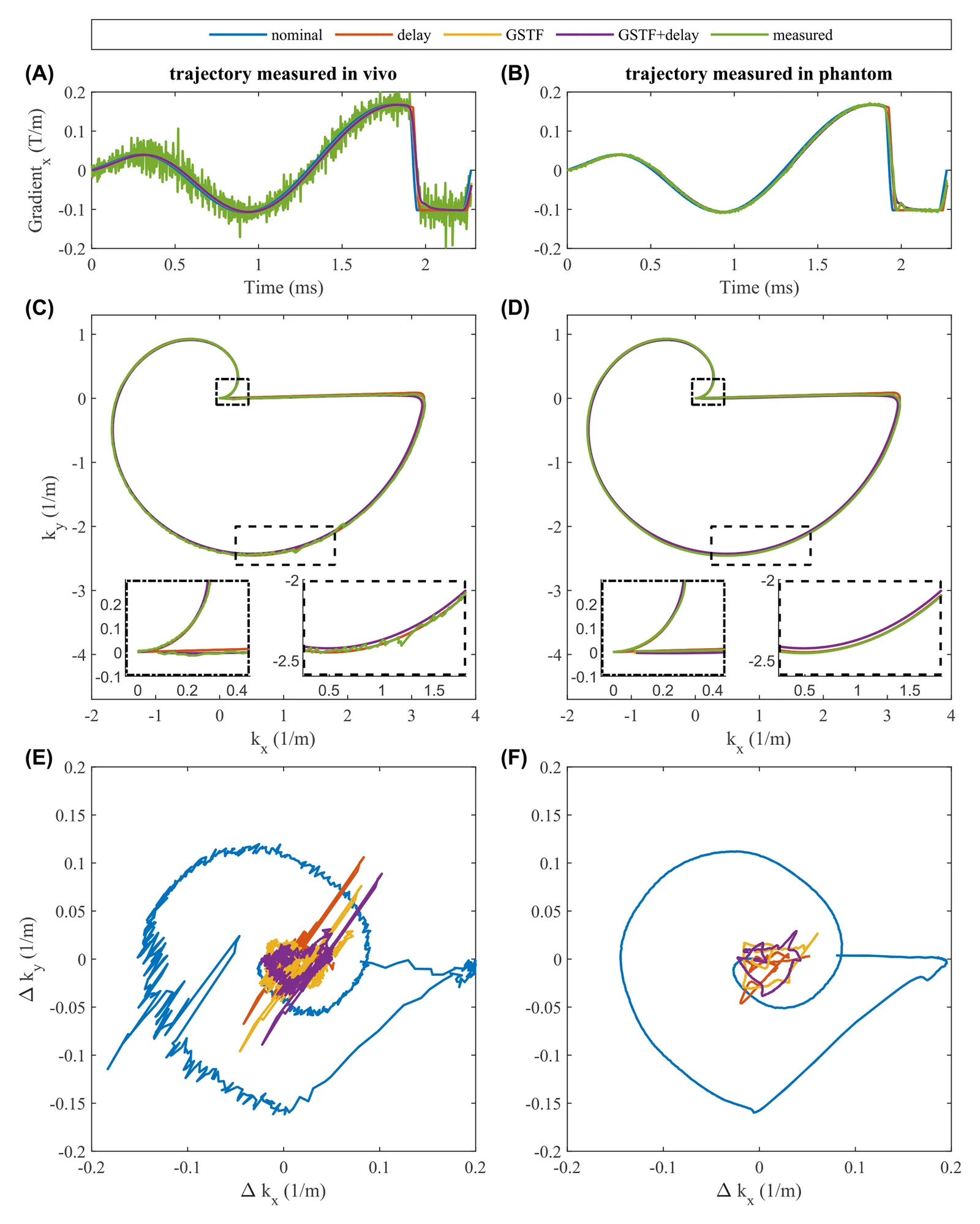
K-space trajectories of one of 96 spiral interleaves of the mouse measurement. Left column: trajectory was measured in vivo. Right column: trajectory was measured in the spherical phantom. (A, B) nominal, corrected, and measured gradient waveform on the x-axis of the gradient system. (C, D) nominal, corrected, and measured k-space values. (E, F) differences of the nominal and corrected trajectories to the measured ones.

Figure 9 shows the gradient waveform on the *x*-axis and the trajectory of one interleaf of the spiral with 96 interleaves that we used in the in vivo experiment. In the left column, the trajectory was measured in the mouse itself, but without any gating or triggering. On the right, the trajectory was measured in the spherical water phantom. Notably, the gradient waveform is much noisier when measured in the mouse (Figure 9(A)) than in the phantom (Figure 9(B)). The trajectory measured in vivo (Figure 9(C)) is not as noisy as the gradient, because the integration has a smoothing effect. However, the zoomed-in insets reveal that there is noticeable noise in the trajectory, which is not the case with the trajectory measured in the phantom (Figure 9(D)). Figure 9(E,F) display the differences of the nominal, delay-corrected, GSTF-, and GSTF + delay-corrected trajectories to the respective measured trajectory. In Figure 9(E), the noise of the trajectory measured in vivo also becomes apparent, since the nominal and the corrected trajectories are noise-free. As illustrated in Figure 9(F), the additional delay in the GSTF + delay correction causes little change in the difference from the true trajectory compared to the GSTF-correction. This aligns with the visual impressions from the images in Figure 8. Both the GSTF-corrected and the GSTF + delay-corrected trajectories have an RMSE from the trajectory measured in the phantom of 0.024 m^−1^. The delay-corrected trajectory deviates less from the measured one, with an RMSE of 0.014 m^−1^.

## 4 Discussion

This study examined different correction methods for spiral k-space trajectories in preclinical MRI. The current gold standard of measuring the trajectory separately in a phantom was compared to an isotropic delay-correction and two GSTF-based correction approaches. We tested three different spiral trajectories in phantom experiments, and one of them also in cardiac in vivo images. In addition to the experimental validation, we also examined the correction methods in numerical phantom simulations.

Evaluating the triangular gradient pulses we used for the GSTF measurements, we discovered that the measured gradient waveforms exhibit asymmetrically occurring undulations, which are not represented by the GSTF (cf. Figure 1(E)). This indicates that the assumed linearity is violated by our high-performance gradient system. Measuring the triangular gradients with different slew rates confirmed this conclusion (data not shown). The undulations were also observed in the gradient waveforms of the spiral imaging experiments (cf. Figure 5(C,E)). Since they cannot be described by an LTI model, both the delay- and the GSTF-based trajectory corrections caused deviations from the true trajectories. This was also reflected in the resulting images, especially for the spiral with 16 interleaves. Relevant LTI violations have already been described for clinical MR scanners.^21,41–44^ However, we demonstrate that the effects appear to be more pronounced on preclinical small-animal MRI systems, than on clinical, human devices. Consequently, we conclude that LTI-based trajectory corrections, in general, cannot achieve the same image quality as measuring the actual trajectory in a phantom. However, our experiments suggest that delay- or GSTF-based corrections can nevertheless be useful in preclinical spiral imaging under specific circumstances.

For the spiral with 96 interleaves, the isotropic delay correction already resulted in an almost as sharp image as the reconstruction with the measured trajectory. For the spiral with 16 interleaves, however, this was not the case. Since the phase of the GSTF of the *z*-axis is quite different from the GSTFs of the *x*- and *y*-axis, it is to be expected that an isotropic delay correction is not optimal for a double-oblique image acquisition. It may work for transverse slices on our scanner, since the GSTFs of the *x*- and *y*-axis have similar phases, but for every other slice orientation, the gradients on the different axes must be corrected with different delays. However, we did not carry out such a correction, because optimizing three individual delays in the same way we did would require at least 11^3^ reconstructions per trajectory (i.e., 11 per axis and trajectory). Since we acquired double-oblique images, but applied the same delay to all three axes, the image quality is deteriorated in the spiral acquisition with 16 interleaves. This phase difference between the different gradient axes is automatically accounted for by the GSTF-based trajectory corrections. It is possible, though, that the actual delays in the spiral acquisitions are slightly different from the delays represented by the GSTF, due to nonlinear behavior of the gradient system. This may be a reason why we still need an additional delay with the GSTF, as will be explained later on. For the spiral with three interleaves, the main effect of the delay correction is a rotation of the object into the correct orientation. The rotation comes from the k-space data being shifted along an arc when the spiral gradients exhibit a delay. This is also the case for the spirals with 96 and 16 interleaves, but the effect is less noticeable because of the shorter readouts.

Another shortcoming of the delay correction is that it does not consider the low-pass characteristic of a gradient system. This may be of little relevance for gradient waveforms that concentrate their energy at low enough frequencies, where the magnitude of the GSTF is close to 1. This is the case for the spirals with 96 and 16 interleaves (cf. Figure 1(J)). Otherwise, it will lead to trajectory errors and therefore image artifacts. In the spiral images with three interleaves, for example, which has higher main frequencies (cf. Figure 1(J)), the phantom appeared too small (cf. Figures 3(F) and 4(F)). We have included a simulation demonstrating this effect in the shared code repository. Investigating whether this geometric misrepresentation impacts quantitative measures, such as functional cardiac parameters, e.g., ejection fraction or stroke volume^1^ should be considered in future studies.

Although the GSTF-based trajectory correction accounts for both the varying gradient delays on different axes, and the low-pass characteristic of the gradient system, the resulting phantom images in our experiments still suffered from artifacts such as blurring, intensity variations, and halos. These are likely caused by the asymmetric discrepancies between the GSTF-predicted and the actual gradient waveforms we described above, since the images reconstructed with the latter hardly show these artifacts. Other possible sources that are known to corrupt spiral images include static off-resonances,^45–48^ eddy current effects of 0th^49^ or higher order,^50^ concomitant fields,^51,52^ or potentially linear cross-responses between the gradient axes. We suppose that the trajectory deviations are the dominant effect in our experiments, for the following reasons: Static off-resonances would have affected the GSTF-corrected images as well as the ones reconstructed with the measured trajectories, but the mentioned artifacts are only present in the GSTF-corrected images (the measured trajectories do not contain spatially resolved information about B_0_ inhomogeneity in the imaged slice). Indeed, we did not apply any correction other than the trajectory measurement to our ground truth images, so all differences between them and the other reconstructions can be assumed to trace back to trajectory differences. We did, however, investigate the effect of zeroth-order eddy currents, which did not alter the images significantly (data available in the shared code repository). Concomitant field effects are expected to be negligible at 7 T: for all three trajectories, the maximum concomitant field contribution of lowest order^52^ at *x* = *y* = *z* = 1cm away from isocenter was 3 orders of magnitude smaller than the maximum field deviation resulting from the gradient deviations between corrected and measured gradient waveforms. As far as higher order eddy currents and gradient cross-responses are concerned, it did not deem necessary to correct them, as our simulated experiments with the numerical phantom also suggest that the observed effects are caused mainly by the first order gradient self-terms.

One might argue that a correction of static *B*_0_ inhomogeneity would have increased the overall image quality, especially in the ground truth images. While this may be true, the subject of this study was to assess the effects of different trajectory correction methods, not to optimize image quality per se. We do agree that a static off-resonance correction should be included in the reconstruction pipeline when it comes to real imaging applications. Considering our acquisition parameters, we would expect the spiral acquisition with the longest readout, i.e., the one with three interleaves, to suffer most from *B*_0_ inhomogeneity. We therefore compared the image in Figure 3(O) to (M,N) (data not shown). The difference images exposed only the slight blurring of sharp edges and the different appearance of the air bubble, which are also visible by eye in Figure 3, but no geometric or other deviations. We therefore argue that our assumed ground truth images are an appropriate choice for the intended purpose, i.e., comparing different trajectory correction methods. For the in vivo experiments, the spiral readout is so short that we do not expect relevant off-resonance effects. This claim is supported by the similarity of the reconstruction with the measured trajectory (Figure 8(I)) and the Cartesian acquisition (Figure 8(L)).

We demonstrated that the results of the GSTF correction in terms of image quality can be improved by adding an additional delay, resulting in the GSTF + delay correction. We hypothesize that this additional delay can partly compensate the trajectory deviations of the GSTF-prediction caused by the asymmetric undulations in the actual gradients. For the determination of this additional delay, we used a reference image, i.e., the one reconstructed with the measured trajectory. While it would have been possible to determine delays that minimize differences between the predicted and measured gradient waveforms, or trajectories, we believe that the image-based delay-optimization is the most useful at this point, because it results in the best image quality. Differences in the optimal delay for the same trajectory (i.e., the spiral with 96 interleaves in the phantom vs. in vivo) might be due to small differences in the slice orientation, or due to different shim currents carried by the gradient coils. Between the different phantom images, the slice orientation and shim settings are the same, but the gradient amplitudes and dwell times differ. Since we see LTI violations in our gradient system, it cannot be assumed that gradients with different amplitudes (or slew rates) necessarily exhibit the same delays. It is therefore also possible that the delays in the GSTF measurements differ from the delays in the spiral acquisitions. The dwell time difference may have an impact because it is necessary to interpolate between different time grids during image reconstruction (namely the gradient raster time (8.1 μs) defining the nominal gradient waveforms, the dwell time of the GSTF measurement (1 μs), and the dwell time of the respective spiral acquisition (Table 1)), giving room for small timing errors. Apart from that, the actual gradient delay of the system could also depend on the receiver bandwidth. However, a detailed investigation of the LTI violations was out of the scope of this study.

One limitation of this study is that we based the determination of the optimal delays on the comparison to a reference image, which was acquired with the same spiral trajectory, but required a trajectory measurement. To eliminate the additional effort of measuring the trajectory, a more practical method to optimize the delays is needed. Two possibilities that come to mind are the comparison to some Cartesian reference image, which is potentially acquired in the study of interest anyways, or the optimization of certain features of the image itself, e.g. geometric orientation or sharpness of edges. Finding an objective measure that is independent from separate trajectory measurements should be the goal of future investigations.

Another limitation is that we only describe experiences made with one specific small-animal scanner equipped with one specific high-performance gradient system. Thus, we cannot prove that our assumptions and conclusions necessarily transfer to other systems. This could also be investigated in the future.

Given that the GSTF + delay correction takes into account more imperfections of the gradient system than the isotropic delay correction, one would expect that it generally yields superior results. Instead, we see in Figure 2 that, except for the spiral with three interleaves, the minimum nRMSE of the isotropic delay correction is always slightly lower than that of the GSTF + delay correction. The same holds for the RMSEs we reported for the k-space trajectories of the spirals with 16 (Figure 6) and 96 (Figure 9) interleaves. However, the images reconstructed with the GSTF + delay correction exhibit fewer artifacts at boundaries between different materials (cf. Figures 3(K) and 7(K)) than the isotropically delay-corrected images (cf. Figures 3(E) and Figure 7(E)). In return, the overall contrast between the different materials is less similar to the ground truth images (cf. Figure 4(E,K)), a feature that is reflected more prominently by the nRMSE. We speculate that this is also caused by the nonlinear behavior of the gradient system. A more detailed investigation of the nonlinearities with respect to gradient amplitude, slew rate, zero-crossing, and other variables is required to better understand how they corrupt the GSTF-model. Other nonlinear effects, like thermal heating, have been reported to affect the GSTF on clinical MRI systems,^43,44^ but, to our knowledge, have not been investigated on preclinical scanners.

One possible solution to address LTI violations but still exploit the advantages of a transfer function model has been presented on a clinical scanner by Rahmer et al.^21^ They propose concurrently measuring the output currents of the gradient power amplifiers and using a current-to-field transfer function to infer the true gradient progressions. This would, at least partly, eliminate nonlinear characteristics coming from the amplifiers from the LTI-model. Provided it is technically feasible, it would be interesting to see how this approach translates to a preclinical setup, and/or a setup with high-performance gradients, since both probably use different amplifiers. Another way might be to explore nonlinear models for modelling the gradient system’s transmission behavior, for example, by using machine learning. First steps in the direction of correcting temporal gradient errors this way have already been taken.^53,54^

Even though the LTI-based correction methods we explored in this study could not fully match the image quality reached with the gold standard method, our in vivo experiment represents an instructive example for their usefulness. We saw that the images get very noisy if the measured trajectory is noisy, which is likely to happen in in vivo measurements where uncompensated motion (i.e., caused by cardiac, respiratory movements and/or blood flow) represents an additional confounder. Measuring the trajectory virtually noise-free in a separate phantom experiment requires additional resources. In both cases, the additional measurements are needed for every trajectory and slice orientation. The GSTF, on the other hand, only needs to be measured once and can then be used to correct arbitrary trajectories in arbitrary orientations. A simple delay correction can even be applied without any additional measurements. We expect that these LTI-based methods work reasonably well for trajectories with a short readout, where phase errors cannot accumulate over long periods of time. In these cases, they are both SNR- and time efficient, especially for measurements with several different slice orientations. The demonstrated application in in vivo cardiac imaging represents one example.

## 5 Conclusion

In summary, our study comprehensively evaluated trajectory correction methods in spiral imaging using a preclinical small-animal scanner with a high-performance gradient insert. While measuring the trajectory in a phantom resulted in the highest image quality for all three tested trajectories, we were also able to obtain good images either with a simple isotropic delay correction or with GSTF-based trajectory corrections. We found the reason for remaining trajectory errors to be nonlinearities in the gradients’ switching behavior, which conventional GSTF-based correction models could not incorporate. We therefore conclude:

- If image quality is absolute priority and there are no time constraints, a separate trajectory measurement should be conducted in a phantom.
- If the main frequency of the spiral readout gradients is low enough, and the readout short enough, a simple isotropic delay correction might yield images of similarly high quality.
- In all other cases, a GSTF + delay correction should result in satisfactory images, while saving a lot of time and effort.

Further investigations are required to better model nonlinearities in the gradient switching and to completely eliminate the need for additional phantom trajectory measurements.

## Figures and Tables

**Table 1:** Imaging parameters of the phantom and in vivo measurements. The slight differences in maximum gradient amplitude and slew rate between the phantom and in vivo measurement with 96 interleaves stem from the slight difference in slice orientation.

		Phantom		Mouse	
Trajectory	96 spiral interleaves	16 spiral interleaves	3 spiral interleaves	96 spiral interleaves	Cartesian
TR (ms)	4.8	250	250	4.8	4.8
TE (ms)	0.701	0.701	0.701	0.701	1.429
Readout bandwidth (kHz)	300	200	909.09091	300	131.57895
Readout length (ms)	2.3	10.1	14.0	2.3	1.46
Flip angle	15°	15°	15°	15°	15°
FOV (mm^2^)	30 × 30	30 × 30	30 × 30	30 × 30	30 × 30
Matrix size	192 × 192	192 × 192	192 × 192	192 × 192	192 × 192
Slice thickness (mm)	1.0	1.0	1.0	1.0	1.0
Number of averages	1	1	1	4	2
Max. gradient amplitude (T/m)	0.2235	0.1491	0.6751	0.2331	n.a.
Max. slew rate (T/m/s)	9344.2	8845.4	11895.2	9747.1	n.a.
Samples per interleaf	573	1916	12,488	573	n.a.

Abbreviations: FOV, field of view; TE, echo time; TR, repetition time.

## Data Availability

The measurement data and MATLAB (MathWorks, Natick, MA, USA) code used to generate the results in this work are publicly available at https://zenodo.org/doi/10.5281/zenodo.11258395.

## Abbreviations

ECG: electrocardiogram
FID: free induction decay
GIRF: gradient impulse response function
GSTF: gradient system transfer function
id: inner diameter
LTI: linear, time-invariant/linearity, and time-invariance
nRMSE: normalized root-mean-squared error
NUFFT: non-uniform fast Fourier transform
od: outer diameter

## References

[1] Schneider JE, Cassidy PJ, Lygate C (2003). Fast, high-resolution in vivo cine magnetic resonance imaging in normal and failing mouse hearts on a vertical 11.7 T system. J Magn Reson Imaging.

[2] Meyer CH, Hu BS, Nishimura DG, Macovski A (1992). Fast spiral coronary artery imaging. Magn Reson Med.

[3] Janiczek RL, Blackman BR, Roy RJ, Meyer CH, Acton ST, Epstein FH (2011). Three-dimensional phase contrast angiography of the mouse aortic arch using spiral MRI. Magn Reson Med.

[4] Castets CR, Lefrançois W, Wecker D (2017). Fast 3D ultrashort echo-time spiral projection imaging using golden-angle: a flexible protocol for in vivo mouse imaging at high magnetic field. Magn Reson Med.

[5] Campbell-Washburn AE, Xue H, Lederman RJ, Faranesh AZ, Hansen MS (2016). Real-time distortion correction of spiral and echo planar images using the gradient system impulse response function. Magn Reson Med.

[6] Vannesjo SJ, Graedel NN, Kasper L (2016). Image reconstruction using a gradient impulse response model for trajectory prediction. Magn Reson Med.

[7] Block KT, Frahm J (2005). Spiral imaging: a critical appraisal. J Magn Reson Imaging.

[8] Engel M, Kasper L, Barmet C (2018). Single-shot spiral imaging at 7 T. Magn Reson Med.

[9] van Gorkum RJH, Guenthner C, Koethe A, Stoeck CT, Kozerke S (2022). Characterization and correction of diffusion gradient-induced eddy currents in second-order motion-compensated echo-planar and spiral cardiac DTI. Magn Reson Med.

[10] Tyler A, Lau JYC, Ball V (2021). A 3D hybrid-shot spiral sequence for hyperpolarized imaging. Magn Reson Med.

[11] Allen SP, Prada F, Xu Z (2021). A preclinical study of diffusion-weighted MRI contrast as an early indicator of thermal ablation. Magn Reson Med.

[12] Naresh NK, Misener S, Zhang Z (2020). Cardiac MRI myocardial functional and tissue characterization detects early cardiac dysfunction in a mouse model of chemotherapy-induced cardiotoxicity. NMR Biomed.

[13] Zhong X, Gibberman LB, Spottiswoode BS (2011). Comprehensive cardiovascular magnetic resonance of myocardial mechanics in mice using three-dimensional cine DENSE. J Cardiovasc Magn Reson.

[14] Castets CR, Ribot EJ, Lefrançois W (2015). Fast and robust 3D T1 mapping using spiral encoding and steady RF excitation at 7 T: application to cardiac manganese enhanced MRI (MEMRI) in mice. NMR Biomed.

[15] Gu Y, Wang CY, Anderson CE (2018). Fast magnetic resonance fingerprinting for dynamic contrast-enhanced studies in mice. Magn Reson Med.

[16] Vandsburger MH, Janiczek RL, Xu Y (2010). Improved arterial spin labeling after myocardial infarction in mice using cardiac and respiratory gated look-locker imaging with fuzzy C-means clustering. Magn Reson Med.

[17] Liu ZQ, Zhang X, Wenk JF (2019). Quantification of regional right ventricular strain in healthy rats using 3D spiral cine dense MRI. J Biomech.

[18] Tan H, Meyer CH (2009). Estimation of k-space trajectories in spiral MRI. Magn Reson Med.

[19] Spielman DM, Pauly JM (1995). Spiral imaging on a small-bore system at 4.7t. Magn Reson Med.

[20] Nussbaum J, Rösler MB, Dietrich BE, Pruessmann KP (2019). Nonlinearity and thermal effects in gradient chains: a cascade analysis based on current and field sensing.

[21] Rahmer J, Schmale I, Mazurkewitz P, Lips O, Börnert P (2021). Non-Cartesian k-space trajectory calculation based on concurrent reading of the gradient amplifiers’ output currents. Magn Reson Med.

[22] Sakhr J, Chronik BA (2019). Vibrational response of a MRI gradient coil cylinder to time-harmonic Lorentz-force excitations: an exact linear elastodynamic model for shielded longitudinal gradient coils. App Math Model.

[23] Winkler SA, Schmitt F, Landes H (2018). Gradient and shim technologies for ultra high field MRI. Neuroimage.

[24] Dillinger H, Kozerke S, Guenthner C (2022). Direct comparison of gradient Fidelity and acoustic noise of the same MRI system at 3 T and 0.75 T. Magn Reson Med.

[25] Dillinger H, Peereboom SM, Kozerke S (2024). Beat phenomena of oscillating readouts. Magn Reson Med.

[26] Duyn JH, Yang Y, Frank JA, van der Veen JW (1998). Simple correction method fork-space trajectory deviations in MRI. J Magn Reson.

[27] Zhang Y, Hetherington HP, Stokely EM, Mason GF, Twieg DB (1998). A novelk-space trajectory measurement technique. Magn Reson Med.

[28] Eccles CD, Crozier S, Westphal M, Doddrell DM (1993). Temporal spherical-harmonic expansion and compensation of eddy-current fields produced by gradient pulses. J Magn Reson a.

[29] Addy NO, Wu HH, Nishimura DG (2012). Simple method for MR gradient system characterization and k-space trajectory estimation. Magn Reson Med.

[30] Vannesjo SJ, Haeberlin M, Kasper L (2013). Gradient system characterization by impulse response measurements with a dynamic field camera. Magn Reson Med.

[31] Stich M, Wech T, Slawig A (2018). Gradient waveform pre-emphasis based on the gradient system transfer function. Magn Reson Med.

[32] Brodsky EK, Klaers JL, Samsonov AA, Kijowski R, Block WF (2013). Rapid measurement and correction of phase errors from B0 eddy currents: impact on image quality for non-cartesian imaging. Magn Reson Med.

[33] Kersemans V, Gilchrist S, Allen PD (2015). A resistive heating system for homeothermic maintenance in small animals. Magn Reson Imaging.

[34] Schneider JE, Wiesmann F, Lygate CA, Neubauer S (2006). How to perform an accurate assessment of cardiac function in mice using high-resolution magnetic resonance imaging. J Cardiovasc Magn Reson.

[35] Gilchrist S, Kinchesh P, Kersemans V (2021). A simple, open and extensible gating control unit for cardiac and respiratory synchronisation control in small animal MRI and demonstration of its robust performance in steady-state maintained CINE-MRI. Magn Reson Imaging.

[36] Wilm BJ, Dietrich BE, Reber J, Johanna Vannesjo S, Pruessmann KP (2020). Gradient response harvesting for continuous system characterization during MR sequences. IEEE Trans Med Imaging.

[37] Scherzer O, Scherzer O (2015). Handbook of mathematical methods in imaging.

[38] Scholten H, Lohr D, Wech T, Köstler H (2023). Fast measurement of the gradient system transfer function at 7 T. Magn Reson Med.

[39] Fessler JA, Sutton BP (2003). Nonuniform fast Fourier transforms using min-max interpolation. IEEE Trans Signal Process.

[40] Fessler JA Michigan Image Reconstruction Toolbox.

[41] Scholten H, Köstler H (2022). Towards gradient perturbation correction in diffusion weighted imaging based on the gradient system transfer function.

[42] Sommer S, Köstler H, Scholten H (2023). Modelling the gradient system as linear and time-invariant: do the assumptions break down at higher field strength?.

[43] Stich M, Pfaff C, Wech T (2020). The temperature dependence of gradient system response characteristics. Magn Reson Med.

[44] Nussbaum J, Dietrich BE, Wilm BJ, Pruessmann KP (2022). Thermal variation in gradient response: measurement and modeling. Magn Reson Med.

[45] Irarrazabal P, Meyer CH, Nishimura DG, Macovski A (1996). Inhomogeneity correction using an estimated linear field map. Magn Reson Med.

[46] Ahunbay E, Pipe JG (2000). Rapid method for deblurring spiral MR images. Magn Reson Med.

[47] Moriguchi H, Dale BM, Lewin JS, Duerk JL (2003). Block regional off-resonance correction (BRORC): a fast and effective deblurring method for spiral imaging. Magn Reson Med.

[48] Chen W, Meyer CH (2008). Semiautomatic off-resonance correction in spiral imaging. Magn Reson Med.

[49] Robison RK, Li Z, Wang D, Ooi MB, Pipe JG (2019). Correction of B0 eddy current effects in spiral MRI. Magn Reson Med.

[50] Wilm BJ, Barmet C, Gross S (2017). Single-shot spiral imaging enabled by an expanded encoding model: demonstration in diffusion MRI. Magn Reson Med.

[51] Lee NG, Ramasawmy R, Lim Y, Campbell-Washburn AE, Nayak KS (2022). MaxGIRF : image reconstruction incorporating concomitant field and gradient impulse response function effects. Magn Reson Med.

[52] King KF, Ganin A, Zhou XJ, Bernstein MA (1999). Concomitant gradient field effects in spiral scans. Magn Reson Med.

[53] Liu Q, Nguyen H (Mark), Zhu X (2022). Gradient waveform prediction using deep neural network.

[54] Martin JB, Banik R, Does MD, Harkins KD (2024). Gradient preemphasis predicted by reinforcement learning.

